# Epigenetic Biomarkers for Environmental Exposures and Personalized Breast Cancer Prevention

**DOI:** 10.3390/ijerph17041181

**Published:** 2020-02-13

**Authors:** Hannah Lui Park

**Affiliations:** Department of Epidemiology, UC Irvine School of Medicine, Irvine, CA 92617, USA; hlpark@uci.edu

**Keywords:** breast cancer, environmental exposures, lifestyle factors, epigenetics, DNA methylation, transgenerational inheritance

## Abstract

Environmental and lifestyle factors are believed to account for >80% of breast cancers; however, it is not well understood how and when these factors affect risk and which exposed individuals will actually develop the disease. While alcohol consumption, obesity, and hormone therapy are some known risk factors for breast cancer, other exposures associated with breast cancer risk have not yet been identified or well characterized. In this paper, it is proposed that the identification of blood epigenetic markers for personal, in utero, and ancestral environmental exposures can help researchers better understand known and potential relationships between exposures and breast cancer risk and may enable personalized prevention strategies.

## 1. Introduction

While 5%–10% of breast cancers are due to genetic mutations and an additional ~10% are considered familial, most breast cancers are thought to be due in large part to environmental and lifestyle factors (which will be collectively referred to as “exposures”) [1,2,3,4]. However, while a variety of risk factors have been identified to be associated with breast cancer risk, the associations are generally weak and ill-defined. Exceptions to this are exposures that occur infrequently in the population, for example, women who received radiation therapy to the chest area for the treatment of Hodgkin’s lymphoma before age 30, who have a five-fold increased risk [5], and women whose mothers took diethylstilbestrol (DES) or were exposed to high levels of DDT while pregnant with them, which were associated with approximately a 2-fold and 4-fold increased risk, respectively [6,7,8]. 

More common exposures that have been linked to breast cancer risk, including hormone therapy, obesity, alcohol consumption, and insufficient physical activity [9], generally have relative risks less than 2.0, meaning that exposed individuals have less than a 2-fold higher risk compared to unexposed individuals [4]. Nonetheless, this type of knowledge enables potential risk reduction by lifestyle modification. For example, a study by the Women’s Health Initiative (WHI) showed that among postmenopausal women, those who lost ≥5% of their body weight had a 12% decreased breast cancer risk compared to those with stable weight over a mean follow-up time of 11.4 years [10]. 

Despite our current knowledge about various risk factors and the development of risk models which estimate one’s risk of being diagnosed with breast cancer in the next 5 or 10 years, it is not known which individuals who are exposed to a given exposure will actually develop the disease; that is, the discriminatory accuracies of current risk models, especially those that incorporate modifiable factors, are moderate, at best [11,12]. This predicament of uncertainty makes prevention strategies generally difficult to implement as the health belief model theory of health behavior states that health-seeking behavior is influenced by one’s perception of a threat. That is unless the perception of an exposure (e.g., obesity) is viewed by an individual as a sufficient threat, they are less likely to engage in behavior to counter the threat (e.g., lose weight) [13]. Thus, it is reasonable to argue that a better understanding of how exposures mechanistically affect breast cancer risk and who is most susceptible to the exposures would increase an at-risk individual’s willingness to adopt risk-reducing strategies. In addition, individuals who do not have known risk factors can still be diagnosed with breast cancer, suggesting that there are additional risk factors for breast cancer that have not yet been discovered or well characterized.

As most breast cancers are not hereditary, identification and characterization of additional risk factors, especially exposures, and their potential interactions with genetic factors, are needed for a better, more comprehensive understanding of breast cancer risk in individuals, which would enable personalized breast cancer prevention. This paper discusses strategies for improving our ability to identify and characterize such risk factors, the first step being the identification of epigenetic biomarkers for exposures that can be used to estimate individuals’ environmental exposures in large cohort studies with archived blood DNA and long follow-up time.

### 1.1. Towards a Better Understanding of how Exposures Affect Personal Breast Cancer Risk

Exposures associated with disease risk are usually identified using data from large prospective cohort studies with years of follow-up or case-control studies. However, retrospective self-reports are vulnerable to recall bias, and most prospective studies did not collect information on timing, duration, intensity, or frequency of exposures which may be of interest to current researchers [14,15]. Even if questions had been asked *a priori*, exposure misclassification is still possible, intended or unintended. For example, studies have found that about 20% of pregnant women with cotinine levels (which objectively indicate nicotine use up to the past 144 h [16]) indicative of current smoking failed to report their smoking habits when asked [17,18]. For most other environmental exposures, such as air, water, or food pollution, individuals may genuinely have no idea if they were exposed, and if so, the timing, duration, intensity, or frequency of the exposure.

As a result, there are numerous environmental exposures that have been implicated in breast cancer risk but not well characterized, including bisphenol A (BPA), perfluorooctanoic acid (PFOA), phthalates, other industrial chemicals, light-at-night, hormones in food, medications, personal care products, pesticides, and diet. The mechanisms by which these exposures have been linked to carcinogenesis have mostly been studied in animal or cell culture models and include endocrine disruption, chronic inflammation, and epigenetic dysregulation (reviewed in [19,20]). However, without reasonably accurate levels of humans’ exposures to a factor of interest and perhaps the timing of the exposures over their lifetime, their potential relationships with breast cancer risk cannot be properly evaluated. Thus, there is a need for objective biological markers, or biomarkers, for environmental exposures. Objective evaluation of environmental exposures, especially in the context of genetic variations, would enable the development of personalized prevention strategies.

Environmental exposures are usually measured in urine and blood products (i.e., serum/plasma), with a preference for urine for many chemicals including polycyclic aromatic hydrocarbons, pesticides, and phthalates [21,22]. However, many large cohort studies that have finished enrollment and have years of follow-up do not have archived blood and urine samples, while DNA samples are often available [23,24,25,26]. Although the majority of DNA samples were extracted with the intention of identifying genetic variations like single nucleotide polymorphisms (SNPs) and gene-environment interactions for health outcomes, DNA samples can also be analyzed for DNA methylation, which is responsive to lifestyle and environmental factors and may even act as a mechanism of epigenetic memory for environmental stressors [27,28,29]. As such, DNA methylation could serve as potential biomarkers for exposures, which would thus enable the study of exposures on health in long-term cohorts even if exposures data, blood, and urine samples are not available.

### 1.2. DNA Methylation as Molecular Markers of Environmental Exposures

DNA methylation is a type of epigenetic mechanism. Epigenetics, or the study of stable but reversible changes in DNA that can affect gene expression but do not involve changes in the DNA sequence, may be key in understanding how environmental exposures affect an exposed individual’s disease susceptibility [30]. For many genes, DNA methylation patterns are cell and tissue-specific and can be highly dynamic in normal differentiation, development, aging, disease, and in response to environmental exposures [31]. For example, cancer cells generally exhibit global DNA hypomethylation and promoter hypermethylation, which can lead to genomic instability and silencing of tumor suppressor genes [32,33,34]. More than 70% of breast cancers exhibit promoter hypermethylation in at least one among a panel of four genes (*GSTP1*, *BRCA1*, *CDH1*, *p16*) [33].

Most studies examining the potential relationships between environmental exposures and DNA methylation in humans have been done using peripheral blood DNA, given that blood draws are generally viewed as non-invasive and routine by the general healthy population compared to tissue sampling, which is more commonly done when specimens are available from biopsies or surgery from patients being tested or treated for illness. Interestingly, peripheral blood DNA methylation at specific loci in the genome have been shown to be associated with specific exposures and can even serve as objective markers of the exposures, which can be especially helpful in studying exposures prone to recall bias or other uncertainties that frequently accompany self-reported questionnaire data [35]. For example, hypomethylation at several sites, most notably within the *AHRR* (cg05575921) and *F2RL3* (cg03636183) genes, has been consistently associated with cigarette smoking in numerous studies [36], outperforming serum cotinine, a classic biomarker for smoking status. In one study, *AHRR* hypomethylation predicted current smoking status with an area under the curve (AUC) of 0.99 [37]. There were clear dose-response relationships of *AHRR* and *F2RL3* hypomethylation with both current and lifetime smoking intensity, as well as with time since smoking cessation, and it took more than 20 years for a full “recovery” of methylation levels [38,39]. These findings suggest that *epigenetic signatures can represent a biological memory of past exposures* and that such signatures may be retained in the exposed stem and progenitor cells [29]. More recently, AHRR hypomethylation in saliva was also shown to predict smoking with an AUC of 0.971 [40]. Thus, salivary DNA methylation may also serve as biomarkers of environmental exposures.

DNA methylation has been shown to be associated with various other environmental exposures, including aging [41,42], BMI [43,44,45,46,47,48,49], physical activity and inactivity [50,51], air pollution [52,53,54], stress [55,56], depression [57], and alcohol [58,59,60,61] (Table 1). Remarkably, methylation of *CDC42BPB* (cg04987734) was shown to be able to classify individuals with respect to heavy alcohol consumption from non-drinkers with an AUC of 0.88. A combination of this marker with one and three additional CpG markers increased the AUC to 0.95 and 0.98, respectively [59]. In an intervention study, after a mean of 21 days of inpatient enforced abstinence, the methylation status of cg04987734 began to revert to baseline values [62]. In a separate meta-analysis, a set of 144 CpGs discriminated between current heavy alcohol drinkers from non-drinkers (AUC > 0.90) across five cohorts [60]. As a biomarker, this panel of CpGs performed better than commonly used clinical biomarkers in discriminating current heavy alcohol drinking [63].

Several exposure-mediated DNA methylation markers have also been shown to be effective markers of disease risk. For example, Zhang et al. showed that individuals in the lowest quartile of *AHRR* and *F2RL3* methylation had almost 16 and 11 times the risk, respectively, of developing lung cancer [64]. In addition, *AHRR* hypomethylation was also shown to mediate the effects of smoking on bladder cancer risk in postmenopausal women [65]. Further, *AHRR* and *F2RL3* hypomethylation were strongly associated with all-cause, cardiovascular, and cancer mortality, suggesting that methylation at these sites may be potential mediators of the detrimental impact of smoking on mortality [38,66,67]. cg04987734 methylation, the marker for heavy alcohol consumption, was also found to be associated with all-cause mortality [67]. Markers for exposure-mediated breast cancer risk have not yet been identified but are sorely needed. Epigenetic markers may help to distinguish which individuals in an exposed population are more likely to develop disease, which is a severe limitation in our current understanding of how environmental exposures affect breast cancer risk.

The DNA methylation markers described above were identified using white blood cell DNA from peripheral blood samples or saliva samples. From a practicality standpoint, this is important because peripheral blood and saliva DNA samples are non-invasive, generally well accepted and tolerated samples to collect from study participants; furthermore, many cohort studies with long follow-up time already have banked peripheral blood or saliva DNA that can be analyzed for DNA methylation. DNA methylation from archived DNA samples has been shown to be stable even after decades of storage under various conditions [68,69]. Thus, if DNA methylation markers are identified for a given exposure, it is very feasible to conduct association studies on the exposure and breast cancer risk with long follow-up time, using DNA methylation markers as proxies of the exposure of interest.

### 1.3. Towards a Better Understanding of how Exposures Affect Breast Cancer Risk in Offspring and Future Generations

It is also possible that epigenetics can help us understand the potential links between an individual’s environmental exposures and breast cancer risk in their children and even grandchildren. For example, using data from a case-control study nested in a prospective 54-year follow-up of 9300 daughters in the Child Health and Development Studies pregnancy cohort, it was shown that women who were prenatally exposed to high levels of the pesticide DDT had 4 times the risk of developing breast cancer as adults [8]. In the same cohort, an association was found between prenatal exposure to DDT and DNA methylation in blood collected in midlife in *CCDC85A*, *CYP1A1*, and *ZFPM2*, genes which have been previously implicated in pubertal development and breast cancer susceptibility [70]. This example shows that in utero exposure to chemicals can significantly increase breast cancer risk, potentially through epigenetic alterations, and supports the developmental origins of breast cancer [71].

Other examples of in utero exposures that are associated with epigenetic differences have been reported in the literature (Table 1). For example, prenatal smoking has been shown to be associated with DNA methylation variability in cord blood and the placenta, and the variability partially mediated the association of smoking and lower birth weight [72,73,74]. DNA methylation signatures of prenatal smoking were even evident in the peripheral blood of offspring collected during early childhood, adolescence, and adulthood, suggesting that prenatal exposures can have persistent effects on one’s epigenetics [75,76]. Maternal exposure to air pollution was also found to be associated with global and locus-specific changes in DNA methylation in cord blood and the placenta [77,78]. Dutch famine studies have shown that women whose mothers were exposed to famine while pregnant with them had higher body mass index (BMI) and triglycerides (TG) levels compared to women who were not exposed to famine in utero [79,80]. Interestingly, DNA methylation at six CpGs together mediated 80% (95% CI, 38.5 to 100%) of the association between in utero famine exposure and TG levels [81]. There have been reports suggestive of an association between exposure to prenatal famine and breast cancer risk; however, the results were not statistically significant after adjusting for confounders [82,83].

Maternal exposures are believed to be transferred to the developing fetus primarily through the placenta [84]. However, emerging data suggest that exposures in both females and males can affect the health of their children even before conception and embryonic implantation. For example, non-smoking women whose mothers smoked while pregnant with them had larger babies than non-smoking women whose mothers had not smoked while pregnant [85,86]. Women whose mothers smoked while pregnant with them were also found to have children with increased asthma risk [87]. Preconception paternal occupation was found to be associated with brain cancer and leukemia in children [88,89]. Preconception paternal exposure to pesticides has also been shown to be associated with an increase in the rates of hematological malignancies in children [90,91].

Mechanistically, it is thought that germ cells and preimplantation embryos are most susceptible to endogenous and exogenous environmental factors because the epigenome in these cells undergoes dramatic reprogramming [92,93]. While causal links have not been established between individuals’ exposures, epigenetic alterations in germ cells and cancer risk in their children, factors such as dietary folate, alcohol, obesity, smoking, endocrine disrupters, and phthalates have been shown to alter epigenetic marks in germ cells [94,95,96,97,98,99,100]. Animal studies have shown some provocative findings. For example, male rats that were fed a lard-based high-fat diet before and during puberty had female offspring with a higher rate of mammary cancer compared to female offspring of fathers that were fed a corn oil-based high-fat diet. There were significant epigenetic differences in the sperm of the fathers who ate the lard-based diet, some of which were also present in the mammary glands in their female offspring [101].

**Figure 1:**
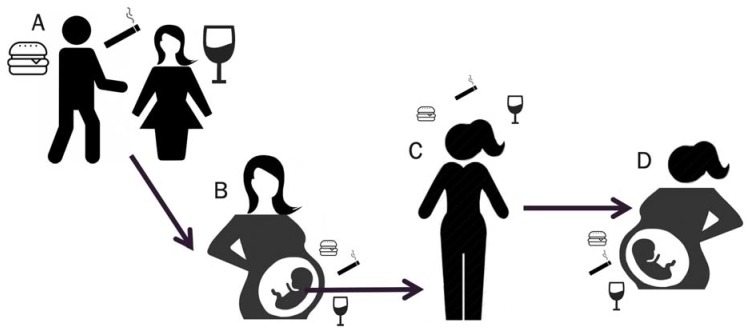
Conceptual model for inherited epigenetic susceptibility to disease. Disease risk in a woman (**C**) may be affected by not only her own lifestyle and environmental exposures but also by what she was exposed to through her mother in utero (**B**) and even through both parents’ pre-conception via exposure-mediated epigenetic changes in their germ cells (**A**). Likewise, the woman’s exposures may affect her future child’s disease risk (**D**).

Maternal exposure to a high-fat diet or synthetic estrogen during pregnancy also increased mammary cancer risk in female offspring and even through several generations [102,103]. Thus, it is possible that analogous to how some individuals may inherit a genetic susceptibility to breast cancer, for example, through inheriting a BRCA1 mutation or breast cancer-related SNPs from their ancestors, some individuals may have inherited an epigenetic susceptibility to breast cancer, resulting from exposures experienced by their parents or even grandparents (Figure 1).

Few human studies have data about intrauterine and parental exposures. Thus, there is a large gap in knowledge about the potential role of in utero and parental environmental exposures and breast cancer risk. Epigenetic markers may enable researchers to close this gap. For example, DNA methylation can function as a marker for prenatal smoke exposure in adults [104,105,106]. One study showed that a prenatal smoking score, derived by combining methylation values from 19 CpG sites, could predict whether the mothers of the adults smoked during pregnancy with an AUC of 0.72105. Identification of additional in utero or pre-conception exposures that are associated with breast cancer risk in humans would present opportunities for breast cancer prevention focused on improving parental environmental exposures and lifestyle during pregnancy and pre-conception.

### 1.4. Towards Personalized Prevention of Breast Cancer

Molecular markers are needed to help distinguish which individuals in an exposed population are more likely to develop breast cancer and which individuals would benefit from undertaking risk reduction strategies such as lifestyle modifications. A better understanding of one’s personal breast cancer risk, perhaps informed by one’s DNA methylation profile in addition to one’s genetic profile, would enable targeted risk reduction strategies. Ideally, an individual’s DNA methylation profile would not only reflect risk markers that they acquired from various personal exposures but perhaps also risk markers they inherited from their parents’ and grandparents’ exposures. On the other hand, it is possible that despite being exposed to various insults, an individual has not acquired the methylation risk markers and is thus not at increased risk. For these individuals, it may not be necessary to undertake risk reduction strategies.

Risk reduction strategies may include trying to reverse the DNA methylation risk markers by undergoing lifestyle modifications (like quitting smoking), medical procedures, or taking medicine. As mentioned previously, the methylation status of the smoking markers *AHRR* and *F2RL3* was highly associated with increased lung cancer risk [64]. However, former smokers whose DNA methylation resembled that of never-smokers did not have an increased risk for lung cancer compared to former smokers whose DNA methylation was consistent with that of current smokers [64]. Although these studies were not longitudinal, they suggest that it is possible to reverse lifestyle-induced epigenetic changes by stopping the lifestyle factor and that this reversal may take longer for some individuals compared to others or not even occur.

Longitudinal epigenetic studies, given their more complicated study design and extended time required, are not as common as cross-sectional ones [162], but some have demonstrated epigenetic changes after a lifestyle intervention, for example, relaxation training [163], physical activity [164,165], and weight loss [96,166]. Interestingly, one of the studies showed that some of the differentially methylated CpGs from baseline to 5 months of weight loss were in the opposite direction as in acquired obesity [166]. In addition, a study in men showed that surgery-induced weight loss was associated with changes in their sperm, especially at genetic locations controlling appetite [96], suggesting a mechanism for markers of obesity (or weight loss) to be passed down to the next generation.

The reversal or absence of an epigenetic marker may signify that an individual is no longer at increased risk for the disease due to that specific exposure, whereas the presence of the marker may signify that the individual is at increased risk for the disease. Knowledge of one’s personal risk for breast cancer, especially if the reliability of the risk prediction is high, is a major factor for determining their willingness to undertake lifestyle modifications to lower their risk [167]. Knowledge that an individual has an inherited or acquired epigenetic breast cancer risk marker that was caused by an exposure can be a powerful motivator for the individual to avoid or counteract the offending exposure.

Animal studies have also shown that epigenetic risk can be reversed by interventions, for example, diet. As a most striking example, it has been shown that maternal supplementation of rats with methyl donors like folic acid or the phytoestrogen genistein can reverse DNA hypomethylation induced by BPA [168]. Pharmacological strategies are also being explored, such as the use of HDAC inhibitors and DNMT inhibitors, which are already being used to treat some lymphomas and solid cancers including breast cancer [169]. However, current epigenetic drugs lack CpG site-specificity, resulting in off-target effects; thus, more research is needed to develop targeted strategies to reverse epigenetic risk markers [170].

Collectively, epigenetic markers of breast cancer risk can be combined with current risk prediction models like the Gail, BCSC, and Tyrer–Cuzick models and their iterations to potentially increase their discriminatory accuracies. Polygenic risk scores have been shown to increase AUCs by up to 0.11 [171,172]. The addition of an Epigenetic Risk Score may increase AUCs even more and thus be more accepted clinically.

## 2. Proposed Approaches and Expected Results

*Challenge #1:* How can we identify epigenetic markers for additional personal exposures that may be associated with breast cancer risk?

Although DNA methylation loci associated with numerous exposures have been identified, most have not been demonstrated to be able to act as markers. The exceptions, CpG markers for current smoking and current heavy alcohol intake, were identified in cohorts that used questionnaires that assessed detailed current and previous use, including type, quantity, frequency, and duration of use, age when first started using, and age when quitting (if applicable) [37,40,59,60]. Identification of epigenetic markers for additional personal exposures that may be associated with breast cancer risk would also require a robust characterization of the exposure and data analysis for potential associations with the peripheral blood epigenome.

Given the relative unreliability of self-reported exposures, especially for those that study participants may not be aware of, such as air, food, or water pollution, and other environmental contaminants, supplementing questionnaire data with biochemical analyses of biospecimens such as saliva, urine, or serum or plasma isolated from blood samples that were collected around the time or from the same blood sample as the DNA that is used for the epigenome analysis, would be ideal to minimize measurement error for the levels of exposure. However, this approach would require funding for the significant costs of not only epigenome-wide analysis (the most commonly used platform being the Infinium DNA methylation arrays, which are in their 3rd iteration, able to analyze >850,000 CpG sites [173]) but also the biochemical analysis (the method of choice is usually mass spectrometry [174,175]) of paired samples, assuming they are available. Besides urine and blood as validation matrices, adipose samples can be used to measure exposure to fat-soluble organics, hair and nail levels are usually indicative of past exposure in the weeks to months range, and placenta and cord blood can be used to assess prenatal levels of exposure [77,176]. Other sources of exposures data include medical records, government-run registries or monitoring programs, employment records, and personal monitoring devices. It is possible that researchers can leverage existing data and/or specimens from current or previous cohorts to identify additional epigenetic markers for exposures.

*Challenge #2:* How can we identify epigenetic markers of ancestral exposures that may be associated with breast cancer risk?

Doing a prospective study to identify epigenetic risk markers inherited from ancestors would take decades. Fortunately, there are several existing cohorts with incident breast cancers, comprised of individuals from more than one generation whose data and biospecimens may be suitable for examination. Assessing levels of ancestral exposures to act as the independent variable will be difficult, but estimations can be made for some ancestral exposures by accessing data from medical records, government-run registries or monitoring programs, employment records, or, as in the case of the Dutch famine cohort, significant historical events. Characteristics and exposures of the individuals and their ancestors would be needed to adjust for potential confounders. A list of transgenerational cohorts is provided in a review by Pembrey et al. [177].

*Challenge #3:* Can epigenetic markers be used to predict if an individual will benefit from lifestyle modifications or clinical interventions to decrease their risk?

Intervention studies with long enough follow-up time for incident breast cancers to develop, and pre-intervention (and ideally, post-intervention) DNA samples would be needed to address this challenge. Several cohorts that meet these criteria already exist. Within the group of individuals who completed the intervention, one could compare the DNA methylation profiles between women who developed breast cancer and those who did not.

The proposed studies would result in the identification of new personal, in utero, and pre-conception lifestyle factors and environmental exposures associated with breast cancer risk in humans, a better understanding of the molecular mechanisms by which exposures and gene-environment interactions mediate breast cancer risk, and the ability to better predict which exposed individuals will actually develop breast cancer and which would benefit from lifestyle modifications and other risk reduction strategies. This would present significant opportunities for personalized breast cancer prevention not only for individuals but potentially also for their daughters, granddaughters, and future generations.

## Figures and Tables

**Table 1:** Lifestyle or environmental exposures associated with differential DNA methylation patterns in humans *.

Exposure	Genes Containing Differentially Methylated CpG Sites or Regions	References
**Self**		
Aging	Panel of 353 CpG sites (Horvath method); Panel of 71 CpG sites (Hannum method)	[41,42]
Cigarette smoking	*AHRR*, *F2RL3*, *GPR15*	[36,37,38,39,40]
Alcohol	*CNTN4*, *CDC42BPB*	[58,59,61]
BMI	*ABCG1*, *CPT1A*, *SREBF1*, *HIF1A*	[43,44,46,47,48]
Physical activity	*PPARGC1A*	[107,108]
Air pollution	*FOXP3*, *IFN-γ*, *ICAM-1*, *TLR-2*	[109,110,111,112,113,114,115,116]
Stress	*BDNF*, *KITLG*, *NR3C1*, *FKBP5*, *MAD1L1*, *HEXCD*	[117,118,119,120,121,122,123,124,125,126,127,128,129,130,131,132]
Depression	*BDNF*, *SLC6A4*, *NR3C1*	[133,134,135,136,137,138,139,140,141,142,143,144,145,146,147]
**In utero/Peri-conception (maternal exposures)**		
Cigarette smoking	*AHRR*, *CYP1A1*, *MYO1G*, *GFI1*	[72,74,75,76,104,105,106,148,149]
Alcohol (in context of Fetal Alcohol Syndrome)	*HLA-DPB1*, *FAM59B*, *CAPN10*, *DES*, *SLC6A3*, *SLC38A2*, *FAM24A*, *H19*, *TGFB1I1*, *PCDHB18*, *PCDHGA*	[150,151]
BMI	*CDHR3*, *ACTL10/NECAB3*, *POM121L1P*, *VIPR2*, *AGRN*, *GGTLC1*	[152]
Air pollution	*COLEC11*	[153]
Stress	*NR3C1*	[154,155]
Antidepressant use	*ZNF575*	[156]
Interpartner violence	*NR3C1*	[157,158]
Famine	*INSR*	[159,160]
Folic acid	*ZFP57*	[161]
**Pre-conception (paternal exposure)**		
Cigarette smoking	*MAPK8IP3*, *TKR* (in paternal sperm)	[97]

* Only studies that identified statistically significantly differentially methylated CpG sites or regions (after adjusting for multiple comparisons) in at least two independent cohorts or in a meta-analysis are referenced in this table. For some exposures, most notably BMI, smoking, and alcohol consumption, there were more genes with differentially methylated CpG sites or regions than listed in the table. Such genes can be found in the references indicated.
